# Assignment of Epidemiological Lineages in an Emerging Pandemic Using the Pangolin Tool

**DOI:** 10.1093/ve/veab064

**Published:** 2021-07-05

**Authors:** Áine O’Toole, Emily Scher, Anthony Underwood, Ben Jackson, Verity Hill, John T McCrone, Rachel Colquhoun, Chris Ruis, Khalil Abu-Dahab, Ben Taylor, Corin Yeats, Louis Du Plessis, Daniel Maloney, Nathan Medd, Stephen W Attwood, David M Aanensen, Edward C Holmes, Oliver G Pybus, Andrew Rambaut

**Affiliations:** Institute of Evolutionary Biology, University of Edinburgh, United Kingdom; Institute of Evolutionary Biology, University of Edinburgh, United Kingdom; The Centre for Genomic Pathogen Surveillance, Big Data Institute, University of Oxford, United Kingdom; Institute of Evolutionary Biology, University of Edinburgh, United Kingdom; Institute of Evolutionary Biology, University of Edinburgh, United Kingdom; Institute of Evolutionary Biology, University of Edinburgh, United Kingdom; Institute of Evolutionary Biology, University of Edinburgh, United Kingdom; University of Cambridge, United Kingdom; The Centre for Genomic Pathogen Surveillance, Big Data Institute, University of Oxford, United Kingdom; The Centre for Genomic Pathogen Surveillance, Big Data Institute, University of Oxford, United Kingdom; The Centre for Genomic Pathogen Surveillance, Big Data Institute, University of Oxford, United Kingdom; Department of Zoology, University of Oxford, United Kingdom; Institute of Evolutionary Biology, University of Edinburgh, United Kingdom; Institute of Evolutionary Biology, University of Edinburgh, United Kingdom; Department of Zoology, University of Oxford, United Kingdom; The Centre for Genomic Pathogen Surveillance, Big Data Institute, University of Oxford, United Kingdom; School of Life & Environmental Sciences and School of Medical Sciences, University of Sydney, Australia; Department of Zoology, University of Oxford, United Kingdom; Institute of Evolutionary Biology, University of Edinburgh, United Kingdom

**Keywords:** SARS-CoV-2, genomic surveillance, phylogenetics, software, lineage

## Abstract

The response of the global virus genomics community to the SARS-CoV-2 pandemic has been unprecedented, with significant advances made towards the ‘real-time’ generation and sharing of SARS-CoV-2 genomic data. The rapid growth in virus genome data production has necessitated the development of new analytical methods that can deal with orders of magnitude more genomes than previously available. Here we present and describe pangolin (Phylogenetic Assignment of Named Global Outbreak Lineages), a computational tool that has been developed to assign the most likely lineage to a given SARS-CoV-2 genome sequence according to the Pango dynamic nomenclature scheme described in Rambaut et al. (2020). To date, nearly two million virus genomes have been submitted to the web-application implementation of pangolin, which has facilitated the SARS-CoV-2 genomic epidemiology and provided researchers with access to actionable information about the pandemic’s transmission lineages.

## Introduction

The response of the global virus genomics community to the COVID-19 (SARS-CoV-2) pandemic has been unprecedented, with a concerted and cooperative effort toward the generation and timely sharing of large numbers of SARS-CoV-2 genomes. At the time of writing, more than 1.8 million SARS-CoV-2 genomes have been submitted to GISAID from over 180 different countries, and this number continues to grow (Figure 1a). The rapid sharing of high volumes of virus genomes has given public health bodies potential access to actionable data as the pandemic has unfolded. This contrasts with the generation and application of virus genomes during some previous global health public emergencies, such as the West African Ebola virus epidemic and Zika virus in the Americas, during which sequencing was more retrospective and undertaken on a smaller scale (Rota et al 2003, Dudas et al 2017, Faria et al 2017, Worobey et al 2019). The rapid generation and sharing of thousands of SARS-CoV-2 genomes, sampled longitudinally as virus transmission has unfolded worldwide, has created an urgent need for accessible tools and systems for managing and interpreting this vast data resource.

In April 2020, Rambaut et al. (2020) proposed and developed a dynamic nomenclature system to name and track global transmission lineages of SARS-CoV-2. This is called the Pango nomenclature (Rambaut et al. 2021) and complements two other SARS-CoV-2 nomenclature systems (NextStrain and GISAID) that focus on broader phylogenetic 'clades', and which incorporate criteria for minimum prevalence and persistence. The Pango lineage nomenclature system is hierarchical and fine-scaled, and designed to capture the leading edge of pandemic transmission. Each Pango lineage aims to define an epidemiologically-relevant phylogenetic cluster, for instance an introduction into a distinct geographic area with evidence of onward transmission (Rambaut et al. 2020). Pango lineages are particularly suited to outbreak investigations at national or regional scales. At the time of writing, there are 1,293 Pango lineages, compared to 12 and 9 clades for the NextStrain and GISAID nomenclatures, respectively.

However, the identification and assignment of SARS-CoV-2 phylogenetic lineages is not a trivial problem, due to the huge size and rapid growth of the virus' global genome data set. The high intensity of genomic sampling of SARS-CoV-2, and the relatively low evolutionary rate of the virus compared to some other RNA viruses (phylogeny branches accrue approximately one nucleotide substitution every 2 weeks on average; Duchêne et al 2020), mean that the fine-scaled lineages of the Pango nomenclature system may differ at very few nucleotide positions. This relatively low level of genetic diversity leaves SARS-CoV-2 classification sensitive to data issues such as missing data, laboratory artefacts, and homoplasy (instances where the same mutation has arisen multiple times across a phylogeny). Some estimates of homoplasy rates for SARS-CoV-2 are as high as 30% of variable sites (De Maio et al 2020), and sequencing amplicon dropouts can account for loss of sequence information from some genomic regions.

Consequently, it is not possible to define high-resolution Pango lineages using only the presence or absence of nucleotide changes at a subset of variable sites. Only by using full genome alignments can we estimate the *most likely* placement of a new genome sequence within the global SARS-CoV-2 phylogeny and thereby assign a lineage name to a sequence. However, even with perfect data, estimating a phylogenetic tree containing >500,000 virus genome sequences is a considerable computational challenge, and efforts have been made to provide 'best-practice' solutions for estimating the global SARS-CoV-2 phylogeny (Lanfear 2020, Jackson 2020).

To overcome these challenges, we developed a computational tool, named pangolin (Phylogenetic Assignment of Named Global Outbreak Lineages), to enable access to actionable information from SARS-CoV-2 genomic data. We have continued to adapt this system as the pandemic progressed to account for the rising number of SARS-CoV-2 genome sequences from a growing list of countries (Fig 1a), the accumulation of genetic diversity among sequences (Fig 1b), and the growing number of designated Pango lineages (Fig 1c).

Figure 2 describes the relationship between the Pango nomenclature system, the implementation of that nomenclature in the form of Pango lineage designations, and the software tool pangolin that can be used to assign the most likely lineage to a given sequence. Using the nomenclature system and rules set out in Rambaut et al., 2020, and maintained by the Pango Network (http://pango.network), lineages are regularly designated by manually curating the global SARS-CoV-2 phylogeny. The Pango Network maintains these designations, publishes an up-to-date record of designated sequences (the 'sequence designation list' at https://github.com/cov-lineages/pango-designation; last accessed: 2021-06-29), and responds to lineage requests via the GitHub repository (https://github.com/cov-lineages/pango-designation; last accessed: 2021-06-29). When a novel lineage is designated the sequence designation list is tagged on GitHub with a description of the changes. This list is then used, together with sequence data from GISAID, to train a machine learning model that can be used to assign lineages (Figure 2). This model, called pangoLEARN, is distributed as a dependency to pangolin (https://github.com/cov-lineages/pangoLEARN; last accessed: 2021-06-29). For the end-user, pangolin can be run as a command line tool or by using the pangolin web application which implements a simple 'drag-and-drop' interface (pangolin.cog-uk.io; last accessed: 2021-06-29), seen in Figure 3.

Here, we formally describe pangolin, a computational tool to assign the most likely Pango lineage to one or more SARS-CoV-2 query genomes. Using pangolin, researchers across the globe have been able to retrieve lineage information; as of 27th May 2021, more than 1.8 million genome sequences have been assigned to lineages using the pangolin web application. This has helped public health networks and researchers worldwide by extracting usable and actionable information from the huge volume of available SARS-CoV-2 genomes.

## Methods

*Global SARS-CoV-2 phylogeny estimation*

Whole genome sequences were downloaded from GISAID (full table of GISAID acknowledgements hosted here: cov-lineages.org/gisaid_acknowledgements.html, last accessed: 2021-06-29). Using the tools available at grapevine (https://github.com/COG-UK/grapevine), these sequences were mapped against the canonical SARS-CoV-2 reference genome (Genbank ID: NC_045512.2) using minimap2 v2.17 (Li 2018). Genome sequences were trimmed to the region defined by positions 265 to 29,674, which correspond to the untranslated regions (UTRs), and the missing 5' and 3' UTRs subsequently masked as N's. Early data releases were based on complete trees containing all sequences, constructed with IQ-TREE v1.6.2, using ultrafast (UF) bootstrapping (Minh et al., 2020; Hoang et al., 2018). However, as the number of sequences increased, an "allocate-and-graft" method was adopted. This involved using the previous list of designated sequences to provisionally *allocate* new sequences to the most likely major viral lineage (A, B, B.1 and B.1.1, and later B.1.1.7 and B.1.177). A separate alignment for each of these major lineages was constructed and a tree for each was estimated using FastTree (Price et al 2010), with a representative from the basal polytomy of that lineage as an outgroup. The lineage trees were then *grafted* together to construct the global phylogeny. More recently, this potential circularity during sequence designation has been avoided by inferring a large maximum parsimony tree *de novo* using FastTree (Price et al 2010). At time of writing, we continue to estimate large maximum parsimony trees as a guide and for specific lineage cases build smaller maximum likelihood trees that include the diversity of interest using IQ-TREE v2.0 (with `–blmin 0.0000000001 -m GTR+G -bb 1000` and all other parameters as default; Minh et al., 2020).

*Manual lineage curation*

Using the lineage designation criteria outlined in Rambaut et al (2020), lineages are curated by hand and annotated onto the global phylogeny in the tree visualization software, FigTree [http://tree.bio.ed.ac.uk/software/figtree/, last accessed: 2021-06-29]. As illustrated in Figure 4, sequences that are 95% complete (i.e. <5% of nucleotide sites in coding regions are ambiguous) on GISAID are considered for lineage designation. As such, not all SARS-CoV-2 genomes on GISAID will get a lineage designation, but pangolin can be used to estimate the most likely lineage of those sequences.

*pangoLEARN*

The pangoLEARN model is a machine learning model that uses the sequence designation list and SARS-CoV-2 whole genome sequences as input for training. It is built using sci-kit learn (Pedregosa et al 2011) and can account for the complete genetic diversity of a lineage. While the underlying data being modeled is hierarchical (i.e. phylogenetic), it was not immediately clear to us that the model needed to represent hierarchy in order to provide accurate classifications. We therefore trialled three types of model: logistic regressions, decision trees, and random forests, using the sci-kit learn implementations (Pedregosa et al 2011).

The decision tree and the random forest models were developed in tandem. A decision tree represents a classification task in a way similar to a flow chart. Each node in the tree represents a decision made based on a value, splitting the problem space into finer and finer pieces until a classification is reached. A random forest is an ensemble of decision trees, each built using a subset of the training samples. Classifications are made when each individual tree "votes" on the final decision. Both of these models inherently represent hierarchy. When trained, their structures ought to approximate the structure underlying the training data.

The trained model provides rapid lineage assignments from within pangolin when queried. On a single thread, pangoLEARN can assign 1,000 genome sequences in ~25 seconds. For all pangoLEARN models, the model was trained using sequences and their designated lineages (outlined above). To account for ambiguities in the data and the limitations of heuristic tree search algorithms used, any identical sequences with conflicting manual assignments were removed from the data set prior to training.

When training the models, each sequence was represented as a vector of one-hot encoded nucleotides. Invariant — and thus uninformative — sites in the alignment were discarded. To handle ambiguity due to sequencing errors, unknown nucleotides were imputed with the reference nucleotide from this location. Tagged versions of the trained pangoLEARN model and header files are hosted on github.com/cov-lineages/pangoLEARN alongside associated metadata and lineage curation notes (last accessed: 2021-06-29).

*Lineage assignment using pangolin*

pangolin is a python-based assignment pipeline built using Snakemake (Koster et al 2012). An input fasta file, containing one or more query sequences, is processed if it fulfills the minimum length (default 10,000 bases) and a maximum percentage of N bases in the genome sequence (default 50%) criteria. Each individual query sequence is mapped against an anonymised lineage A genome from Wuhan using minimap2 v2.17 (Li 2018). Genome sequences are trimmed to only the coding region (positions 265 to 29,674) and the missing 5' and 3' regions subsequently masked as N's. These aligned genomes are then assigned the most likely genome using the pangoLEARN model. In a few instances, specific lineages of interest, such as lineage B.1.1.7 and B.1.351, are also assigned using a set of heuristic rules that explicitly check for the presence of certain SNPs, allowing for a controlled level of ambiguity at crucial sites. In its output report, pangolin documents the version of pangoLEARN model and Pango designations used to assign lineages.

*Limit testing of pangolin*

We evaluated pangolin's robustness and ability to assign lineages in cases of excess diversity, varying levels of ambiguity and in the face of novel recombinants. We selected the most complete SARS-CoV-2 genome for each designated lineage as a representative set (n=1,253 from Pango designation v1.1.23). For each representative genome, we simulated diversity from a given genome ranging from 100% to 90% divergence at 0.2% intervals, producing genomes from 100% to 90% identity of the representative. For each divergence interval, we randomly selected a given number of sites and replaced the nucleotide at that position with a random base distinct from the original base (i.e. 1% divergence equates to 299 sites replaced). We repeated this entire process 10 times, generating 625,000 simulated genomes in total, and assigned lineages to these genomes using pangolin v2.3.2 (pangoLEARN release 2021-05-10).

To test pangolin's behaviour in response to increasing ambiguity, we took the most complete sequence for every lineage as a representative (n=1,253) and randomly replaced a given percentage of sites (in the range of 1-100%) with N. All simulated sequences with >25% N content failed to map against the reference, so results are shown only for simulated genomes within the range 1-25% (Figure 7a). This approach tests how pangolin responds to increasing amounts of random missing data. However, in practice, ambiguous or missing sites are often clustered together as amplicon dropouts. Therefore we subsequently simulated a sliding window of triple amplicon dropouts for each representative genome corresponding to the amplicons formed in the ARTIC primer scheme v3 (https://github.com/artic-network/artic-ncov2019; last accessed: 2021-06-29) and ran pangolin v2.3.2 (pangoLEARN release 2021-05-10) on the simulated genomes (n=1,253 X 98 amplicons).

In order to assess how pangolin assigns novel recombinants, we randomly chose 62,650 distinct lineage pairs from a set containing a single representative genome per lineage (n=1,253). From these pairs, we simulated recombination between the pair for positions at 10% intervals across the genome (i.e. positions 2,990, 5,980, 8,970 etc.; Figure 8a). Each pair was included as the 3' and 5' lineage to ensure the simulations were symmetrical. We then ran a naive pangoLEARN model (i.e. not trained on the recombinant sequences) on these simulated recombinants (pangolin v2.3.2, pangoLEARN release 2021-05-10).

**Results & Discussion**

A comparison of the performance and training time of the three model types used in the various pangoLEARN releases between July and October 2020 is shown in Figure 5. The statistics shown are based on 10-fold cross-validation. The first model to be used was multinomial logistic regression, which provides no representation of phylogenetic hierarchy. Each possible classification was associated with a collection of SNPs, and these associations alone were used to assign each query sequence a lineage. This relatively simple model offered acceptable accuracy, practically matching the performance of a maximum likelihood phylogenetic inference approach, whilst providing classifications in seconds instead of minutes. An additional benefit of this model was the intuitive interpretability of the regression coefficients, as each represented how strongly associated each individual SNP was with a particular lineage assignment. However, training time for this approach depends on not only the number of parameters, but also on the number of classes and samples. Consequently, training time for this model quickly became unsustainable (Figure 5b).

To better represent the hierarchical nature of the data, we next trialed the decision tree and random forest models. Figure 5 demonstrates the performances of these two model types. For our data sets and problem, the decision tree and the random forest approaches performed similarly. We ultimately adopted the decision tree model because the decisions it makes are more easily interpreted, whereas it is often very difficult to interpret the decisions made within random forests. Because each constituent of a random forest is built using only a subset of the model's features, each constituent tree is unlikely to resemble the structure of the underlying data. The output of each of these trees is useful only when used together with the rest of the ensemble. In contrast, the decision tree model meaningfully represents the tree structure in a way that can be easily validated. Additionally, we harnessed the tendency of decision trees to overfit in order to make the model more likely to correctly classify smaller lineages whilst another model might overwhelmingly prefer to suggest a larger lineage. If the methods were applied to a virus with more genetic diversity or a different mode of evolution, the relative performances of these methods, and thus our choice of model, may be different. The modularity of our system (Figure 2) provides a useful level of flexibility: for each lineage release, each model can be re-evaluated and the currently best-performing model can be substituted into the framework.

As with many tools for genomic epidemiology developed in response to the COVID-19 pandemic, it has been challenging to maintain sufficiently fast computation times in the context of data sets that have grown exponentially in size. Our experience to date suggests that many kinds of "flat" models (those in which the hierarchical structure of the underlying data is ignored) may well be intractable given the hundreds of lineages, thousands of parameters, and hundreds of thousands of genomes available for analysis.

*Limit testing of pangolin*

In order to test how pangolin performs as sequences become increasingly different from the training data, we simulated a distribution of genomes that were up to 10% divergent at the nucleotide level from a representative of each lineage present in the training dataset (n=625,000 simulated genomes from 1,253 lineage representatives). At 100% identity, pangolin correctly assigns 94% of lineages (Figure 6). Of all simulated genomes, 179,628 genomes were assigned the same lineage as the designation of the genome they had been simulated from ("correct" lineage assignment). Of genomes that were not assigned the same lineage as source genome lineage designation, 154,176 genomes were assigned the parent lineage, 3,791 were assigned a descendant 'child' lineage of the designated lineage and 301,435 genomes were assigned an incorrect lineage. Figure 6a shows that 95% of correct assignments occur when simulated divergence is 98.8% identity or more (i.e. <1.2% divergence from the lineage reference) or 358 nucleotide substitutions across the virus genome. Figure 6b shows there to be a drop off in accuracy when the pangoLEARN model encounters sequences that are very different from what it has been trained on. We are aware of this decline and to compensate for it and minimise its effects we regularly and frequently train new pangoLEARN models with the latest list of designated sequences.

We simulated random ambiguities across SARS-CoV-2 coding regions for a set of representative genomes for all lineages (n=1,253). Figure 7a shows pangolin performs well and is robust to some missing data. As the percentage of ambiguity increases, the number of correct assignments declines, however the proportion of genomes assigned to parent lineages then increases (this is the designed behaviour of our method). We observe a small increase in incorrect assignments, however beyond 24% ambiguity, minimap2 fails to map and therefore a lineage cannot be assigned.

These simulations generate N's randomly across the coding regions of the virus genome. In practice, we often see clusters of ambiguities that reflect low coverage regions or amplicon dropouts in the data, inflating the overall ambiguity percentage, but still allowing minimap2 to successfully map the rest of the genome. We tested this case with a sliding window of triple amplicon dropouts across the genome (Figure 7b shows amplicons corresponding to the ARTIC ncov2019 primer scheme v3 and their genomic location). A total of 126,553 simulated genomes were assigned using pangolin (Figure 7c). Overall, pangolin is robust to amplicon dropouts, however there are some key regions that, when missing, lead to a greater proportion of misassignments (either of an ancestral lineage or an incongruous lineage).

Lastly, we tested pangolin's behaviour in response to novel recombinant genomes made up of two distinct lineages (Figure 8). We randomly chose 62,650 distinct lineage pairs from a set of representative genomes (n=1,253) and recombined them in varying proportions at 10% intervals across the length of the genome (Figure 8a ,b). Each lineage of the pair was recombined as the 5' lineage and the 3' lineage to ensure recombination simulations were symmetrical. We ran 1,378,300 tests of pangoLEARN to assess how it responds to novel recombinants. Intuitively, recombinants highly skewed towards one lineage over the other are more likely to get assigned the dominant lineage (e.g. the 10% 5' recombinant; Figure 8d). As the proportion of the 5' lineage increases, the 5' lineage is more often assigned and the proportion of genomes assigned to an ancestral lineage of the two lineages increases. There is a skew towards assigning the lineage with its 3' end represented, which suggests there may be features at the 3' end of the genome that may be more informative for lineage assignment. Similar to Figure 7c, certain key sites at the 3' end of the genome appear to be important for accurate assignments. We investigated this using the decision tree rules output by the pangoLEARN model (https://github.com/cov-lineages/pangoLEARN; last accessed: 2021-06-29) and Figure 8c shows a density curve reflecting the number of rules within the decision tree model that a given site in the SARS-CoV-2 genome is included in. The large spike towards the 3' end of the genome equates to position 28,882 in the reference genome and is one of the three defining SNPs of the B.1.1 lineage. At present, pangolin cannot detect novel recombinants and can only assign based on previously designated lineages.

*Limitations*

The approach to lineage assignment currently implemented in pangolin provides a responsive, scalable tool. We continue to reflect the latest diversity of SARS-CoV-2 by retraining the pangoLEARN model each week and can assign lineages to the entire GISAID database in a matter of hours. However, our approach has a number of limitations. Unlike phylogenetic assignment, the machine learning approach implemented in pangolin does not natively handle ambiguous data. To allow for nucleotide ambiguities, using an early lineage A reference sequence (accession: EPI_ISL_406801), we impute reference nucleotide states at sites with missing or ambiguous data. However, the reference nucleotide is not equivalent to an N and the consequences of this approach may be to provide more rootward (basal) lineage assignments for sequences with low coverage. As the Pango nomenclature system is hierarchical, a more rootward assignment can be interpreted as a lower-resolution (high taxonomic level) classification rather than an incorrect one (i.e. the sequence could belong to the assigned lineage or any of its descendent lineages). As is common practice in machine learning applications, we treat our training data and query data in the same way. To limit how this imputation may affect the training and therefore the model, we do not allow highly ambiguous sequences in the training set (cut off of 5% ambiguity).

Another consideration of our approach is that, except for particular lineages of concern, the assignments are largely unsupervised. Decisions are being made internally by the trained model and accurate assignment for a given lineage may rely on one or two key SNPS. This can lead to misassignments when incomplete data is queried with pangolin. For example, a sequence that might rightly belong in lineage B.1.177 but which has Ns in lieu of one or two important SNPs would be in fact assigned to a more basal lineage in the tree, such as B.1. We also commonly observe homoplasies in SARS-CoV-2 genome sequence data, which in combination with other mutations may inform assignment decisions for multiple lineages. Theoretically if some key sites are missing, there may be identical sequences informing multiple lineages once their unknown values are imputed. To avoid such conflicts in the training data, we have implemented a 5% ambiguity cut off for the training set (as of Pango designation version 1.2) and any conflicting sequences are identified in a pre-screening process before training, and sequences belonging to a minority lineage are removed.

A further limitation of our approach is that the quality of the machine learning assignments decline as genetic distance from the training set increases, as described in Figure 6. This means that the pangoLEARN model depends on regular updates to the list of Pango designated sequences as input. Pango designation has recently become a more formalised process, with the formation of the Pango Network (https://www.pango.network/; last accessed: 2021-06-29), a team of experts and volunteers from around the world who will work to maintain these lineage designations alongside crowdsourced input through GitHub requests.

*Future directions*

The pangolin tool is being developed as virus transmission and diversification is ongoing, and in the context of rapidly growing numbers of SARS-CoV-2 genome sequences. We are exploring and trialling more sophisticated machine learning models, including a hierarchical logistic regression model. The current pangolin model can accept all designated sequences for training, which now comprises >400,000 genome sequences. In the future it may be necessary to downsample this input. Indeed, as some lineages become unobserved, inactive, and eventually extinct in the global SARS-CoV-2 data set, they can be removed from the training set. Similarly, using a single large global phylogeny may not continue to be practical beyond a million sequences and we may need to consider only more recently sampled genomes, with deeper nodes in the global phylogeny being represented by fewer samples. This approach may have implications for countries not generating or sharing sequence data promptly, or if SARS-CoV-2 diversity in some areas of the world has been poorly characterised or unsampled. In this case, we will be able to retrain our models rapidly and incorporate new virus genetic diversity that may come to light in the future. With the current global push towards real-time data generation and sharing and the growing global genomics capacity, these approaches for exceptionally large data sets will be needed.

Although pangolin is a SARS-CoV-2 lineage assignment tool, the framework it implements could be adapted easily for use in future outbreaks involving another virus. A similar selection process among machine learning models would likely be needed as each virus evolves differently and presents unique analytical challenges. However, the modular toolkit and suite of machine learning models that we have developed and implemented in pangolin will enable it to become a generalised pathogen typing tool. Indeed, for other more established viruses with greater diversity, we predict the pangoLEARN approach would effectively assign virus subtypes.

Since its initial release, pangolin has provided researchers with the ability to easily compare SARS-CoV-2 sequences and gain information to inform public health decision making. Using the associated pangolin web-application and command line tools, phylogenetic information from SARS-CoV-2 genomics is readily accessible. Remarkably, almost 2 million unique sequences have been assigned by the pangolin web-application alone to date. By making the code and the lineage assignments openly available, we invite the broader community to contribute to the growing, dynamic list of SARS-CoV-2 lineages.

## Data availability pangolin is hosted publically on GitHub and available under a GNU General Public License v3.0. The pangolin web application is available at pangolin.cog-uk.io (last accessed: 2021-06-29). Daily updated information about all Pango lineages and links to all resources are hosted at cov-lineages.org.

## Funding

A.OT is supported by the Wellcome Trust Hosts, Pathogens & Global Health Programme [grant number: grant.203783/Z/16/Z] and Fast Grants [award number: 2236]. OGP and LdP acknowledge support from the Oxford Martin School. AR, RC, JTM acknowledge support from the Wellcome Trust (Collaborators Award 206298/Z/17/Z ARTIC network). EC and AR are supported by the European Research Council (grant agreement no. 725422 – ReservoirDOCS).

## Acknowledgements

We thank users who submitted suggestions on the pangolin GitHub repository, with particular thanks to Torsten Seeman and Art Poon for their comments and feedback. We thank Rob Lanfear for discussions and for maintaining a best practice phylogeny. We would also like to extend our gratitude to all involved in the global SARS-CoV-2 genome sequencing effort and continuing to share data on GISAID (https://www.gisaid.org/; last accessed: 2021-06-29, Elbe & Buckland-Merrett 2017), contributing to open science and enabling tools like pangolin to represent the latest diversity of SARS-CoV-2.

**Figure 1**: a. Time series of the number of countries that have reported SARS-CoV-2 genome sequences (shaded area) and a curve showing trends in the geographic variation of reporting, quantified as the Shannon diversity (H) of sequence sampling location labels (curved). b. The accumulation of SARS-CoV-2 genetic diversity over time, measured as the mean genetic distance of sampled sequences from the reference sequence (accession: EPI_ISL_406801). Shaded regions indicate one standard deviation from the mean. c. Number of designated Pango lineages over the course of the pandemic. As more countries have contributed sequences, and as genetic diversity has accumulated throughout, the Pango nomenclature has continued to define distinct lineages that represent the emerging edge of the pandemic. SARS-CoV-2 genome sequences and metadata described were sourced from GISAID on 2021-05-31.

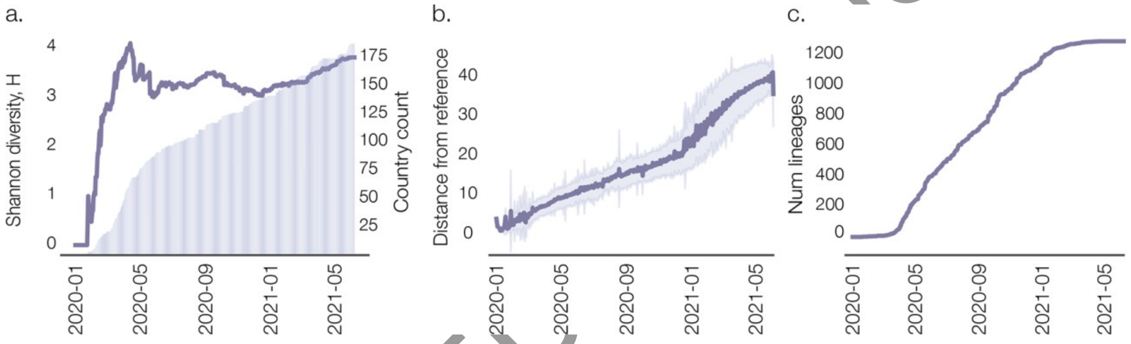

**Figure 2**: Workflow describing the process of Pango lineage designation and assignment of lineages to SARS-CoV-2 genome sequences using pangolin. An estimated global SARS-CoV-2 phylogeny is periodically manually curated to 'designate' lineages and sequences (left). A list of sequences and the lineages to which they have been designated (the "sequence designation list") is maintained by the Pango team at https://github.com/cov-lineages/pango-designation (last accessed: 2021-06-29). These designations and the associated genome sequences from GISAID are used as input for the pangoLEARN training pipeline (https://github.com/cov-lineages/pangoLEARN; last accessed: 2021-06-29) (centre). Once this is completed a new pangoLEARN data release is tagged (centre). This creates the machine learning model that pangolin uses to assign genomes (right). Users can then submit a SARS-CoV-2

genome query sequence and pangolin will assign the most likely lineage based on the currently established lineage designations. * In addition to assignment using the pangoLEARN model, certain lineages of interest are assigned by checking for specific defining SNPs with some built-in flexibility (e.g. B.1.1.7 is assigned by checking for the presence of at least 5 of the 17 defining SNPs that fall on the basal branch of the lineage). These additional ad hoc rules may be subject to revision or removal to maintain performance of the pangolin system.

**Figure 3.** pangolin web application interface**.** a. The image shows the landing page of the pangolin web application where users can either select or drag-and-drop a local file into the web browser. b. The results page showing a processed file, the sequence name for each sequence and the assigned lineage. Links to the UK and global microreact.org builds, as well as the cov-lineages.org web pages for each lineage are represented by the three icons on the right.

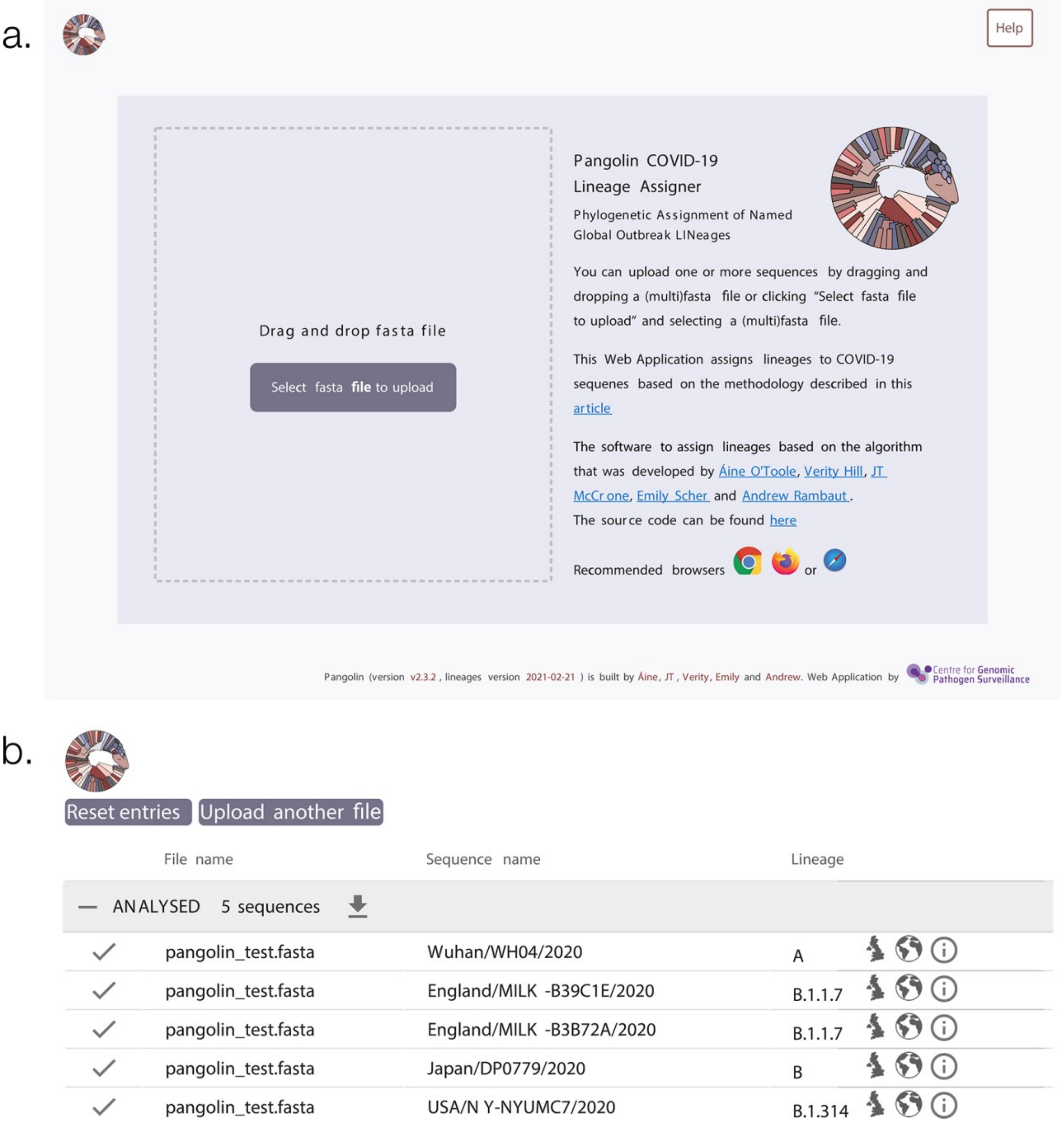

**Figure 4.** Distribution of genome completeness as a percentage of informative coding region sites for all SARS-CoV-2 sequences on GISAID. 1,382,550 sequences were assessed for ambiguity in coding regions, including both whole genome sequences with high ambiguity content and short fragments that have been uploaded to GISAID. 1,284,427 sequences had <5% ambiguous sites across the virus coding region (i.e. were at least 95% complete). Sequences that have designated a lineage are indicated (n=438,440). This 95% completeness threshold was enacted as of Pango designation version 1.2 (GISAID data sourced on 2021-05-07).

**Figure 5.** Performance of different pangoLEARN models. a. Number of genomes submitted to GISAID by the date of model training, and thus number of genomes included in a given model training. b. Training time (hours) for each model type (logistic regression, random forest, decision tree), tested on SARS-CoV-2 genome data releases from April to October 2020. All models except the multinomial logistic regression scale acceptably with increasing sequence and lineage counts. c. The average lineage recall rate for each model for each data release. All models performed well, with the random forests each slightly beating the decision trees. d. The average F1 scores for each of the models for each of the data releases. These scores were closely correlated with the recall rate.

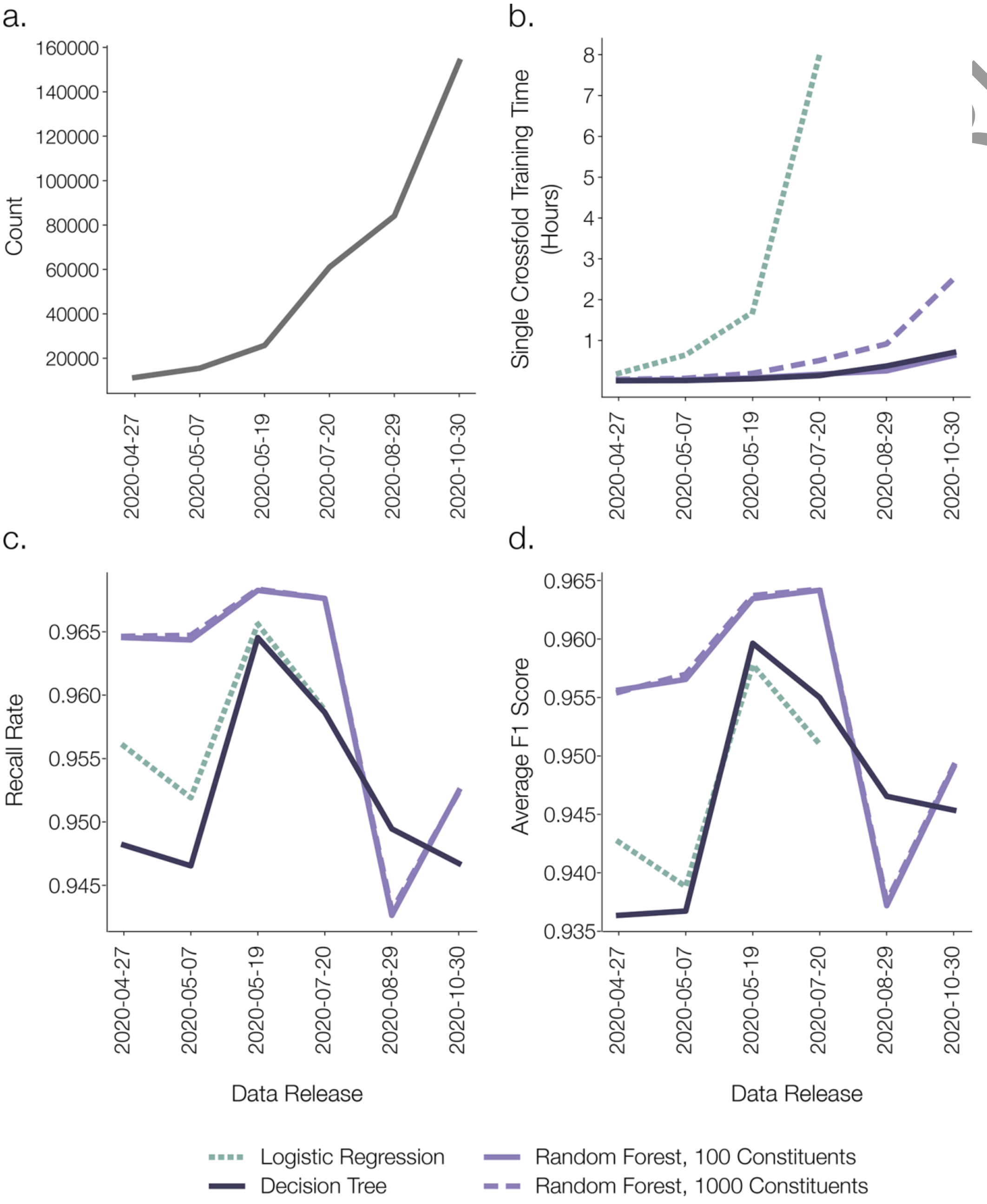

IPT

**Figure 6**: Performance of pangolin for genomes with increasing numbers of simulated additional mutations. a. Boxplot showing the spread of the majority of data, separated by whether the lineage was correctly assigned, assigned an ancestral lineage of the designated lineage, assigned a descendant lineage, or incorrectly assigned. The whiskers define the 5th and 95th percentile range. b. Proportion of genomes assigned correctly, incorrectly or to an ancestral or descendant (child) lineage, normalised for a given percentage identity.

**Figure 7:** Performance of pangolin in response to missing data. a. pangolin assignment accuracy over a gradient of percentage ambiguities. Correct assignments decline with increasing numbers of ambiguous sites, and this initially leads to a greater proportion of genomes assigned to the parent lineage. At greater % of ambiguities, minimap2 fails to effectively map against the reference genome and beyond 24% ambiguity all genomes fail to map. b. Overlapping amplicons generated from the ARTIC ncov2019 primer scheme v3 (98 amplicons across two pools). c. pangolin assignments over a sliding window of triple amplicon dropouts coloured by correct lineage assignment, ancestor lineage assigned or incorrect assignment. SARS-CoV-2 genome schema with genome positions and largest genes (ORF1ab, S, M and N) labelled.

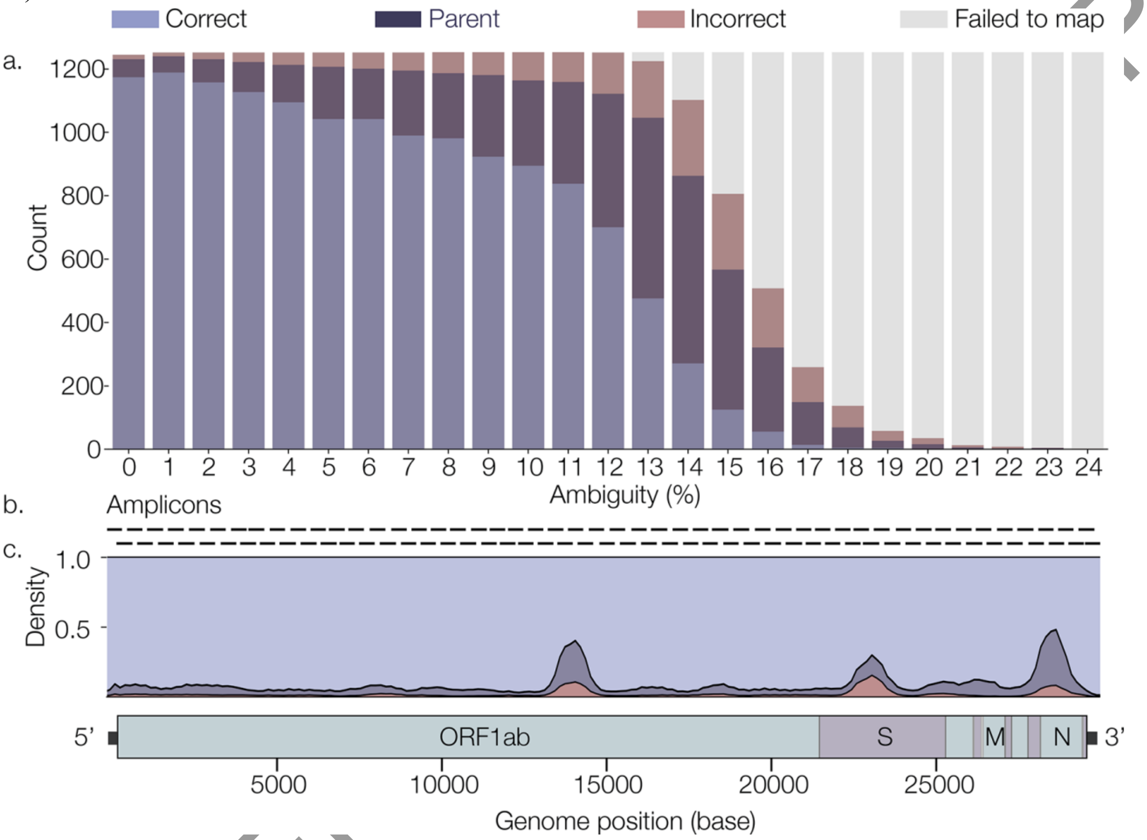

ACCEPTED

**Figure 8:** The behaviour of pangolin in response to simulated recombinant genomes. a. Genome graph of SARS-CoV-2. The position of percentage cut-off sites is shown along the top of the graph and nucleotide base position along the bottom. b. Schema describing the structure of simulated recombinants. Each recombinant is a combination of two distinct lineages in varying proportions. The three example recombinants show a 20% 5' lineage (80% 3' lineage) recombinant, a 50:50 recombinant and an 80% 5' lineage (20% 3' lineage) recombinant. c. A density curve over the SARS-CoV-2 genome highlighting the relative importance of particular sites within the decision tree. The density is calculated based on the number of rules in the decision tree that include a given site in the genome. d. The horizontal axis indicates the percentage of the 5' lineage present in a given recombinant genome. Each bar represents 125,300 simulated recombinants. Stacked colours indicate the count of the recombinants that had either the 5' lineage assigned, the 3' lineage assigned, an ancestral lineage assigned, or an incorrect assignment (i.e. a sibling or unrelated lineage).

RIPT
